# Anæsthetics

**Published:** 1874-12-01

**Authors:** 


### ANÆSTHETICS.

*From The Doctor, Nov. 1, 1874.*

IN our last issue we gave an account of Nelaton's treatment of chloroform narcosis, as related at the Norwich meeting of the British Medical Association in papers by Dr. Marion Sims and Sir J. R. Cormack, M.D.

In the same section (Surgery) there was also read a paper "On the Administration of Laughing Gas and Ether, singly, or combined in any Proportion desired," by Mr. J. T. Clover, whose apparatus possesses great advantages.

The pungency of ether-vapor, when not largely diluted with air, and the frequency of violent delirium when it is so diluted, were spoken of as serious objections to its use. Mr. Clover's apparatus supplied, first, pure laughing gas, which was not unpleasant to breathe; and afterwards, by turning a dial on the face-piece, supplied ether-vapor gradually. This was done with the hand which held the face-piece, and consequently the other hand was at liberty to feel the patient's pulse. Less ether was required than when no gas was used; consequently, less of it was absorbed by the tissues of the body, and recovery from the effects of the anæsthetic took place more quickly and with less intoxication.

Mr. Lawson Tait differed from Dr. M. Sims as to the cause of death from inhaling chloroform being due to cerebral anæmia. He thought it more probable that the explanation lay in some poisoned condition of the blood. There was no evidence to show that death from chloroform ever occurred to an infant or to a woman during labor.

Dr. Humphry thought the administration of chloroform greatly increased the responsibilities of operative surgery; and there were few surgeons who had not experienced that dreadful suspense in cases of chloroform narcosis where death appeared imminent. He thanked Dr. Sims for the additional hint as to what to do in cases such as these. Dr. Humphry thought that allusion had not been made to those cases where death took place at the early part of the administration, preceded with violent convulsions. He considered that such cases could not be attributed to cerebral anæmia, and he agreed with Mr. Lawson Tait that an explanation was to be sought in some altered condition of the blood. He suggested that nerve exhaustion might explain this, violent excitement being followed by correspondingly intense depression. He had observed, in cases of death following upon chloroform narcosis, that the blood was of a consistence like treacle.

Mr. Baker mentioned that he always gave the patient a dose of brandy before proceeding to administer chloroform, with very good effect.

Mr. Lund alluded to the importance of always having at hand, when administering chloroform, means for applying galvanism, and illustrated this by cases which had come under his notice at the Manchester Infirmary.

Sir J. Rose Cormack attached less importance to inversion than Dr. Marion Sims, and thought it would be a dangerous mistake unduly to exalt its efficacy. In cerebral anæmia from any cause, the patient ought, as a rule, to be placed in the horizontal position, with the head low and the feet raised about a foot. The cases in which inversion is called for are rare —quite exceptional. It ought, moreover, to be performed most carefully and watchfully—tentatively, in fact. Too long continued inversion may prove very dangerous by causing distention of the right side of the heart, which will in turn cause and maintain a cessation of its contractions—which causes death in a certain class of cases of chloroform inhalation. This and other poisons, such as creosote and prussic acid, given in large doses, produce instantaneous paralysis of the right side of the heart; the auricle goes on receiving blood and is unable to get rid of it, so that by distention the paralysis (which in some cases would be only momentary) is rendered permanent; the result is probably that instant or very rapid death to which the French apply the term *sideration*—death by star-influence. This subject of quick death from sudden distention of the right side of the heart, he had investigated experimentally many years ago, and he still held to the views he had then published. Touching Mr. Clover's apparatus, it was unquestionably a most admirable invention, but its cost and complexity must prove fatal to its introduction into general use. It will be a great source of safety when the administrator is a steady, watchful man, familiar with the system and the instrument. On the other hand, it is apparent at a glance that negligence or hardihood might readily cause an untrained or self-sufficient administrator to turn the valves too much, too little, or not at all. The induction of complete anæsthesia for surgical operations can never be divested of all danger; but (in theory at least) by Mr. Clover's method the danger may, it would appear, be reduced to its minimum. Slow administration, using the conically arranged towel, and letting plenty of air be breathed, generally secure immunity from danger; and, under all circumstances, such precautions and appliances can be commanded. When cost and complexity are not objections, and when Mr. Clover's apparatus and a person who knows well its use are at hand, it ought to be employed. The remark which was made by Mr. Baker was of great importance—viz., that a moderate stimulant, a dose of wine or brandy, given shortly before the commencement of inhalation, greatly tends to prevent the anæsthetic vapor from producing a too depressing influence on the heart.

### THE ACTION OF ANÆSTHETICS ON THE RED CORPUSCLES OF THE BLOOD.

In connection with the papers above noticed, it may be well to draw attention to Professor Huter's views, as stated in an article on stasis and embolism of the blood-corpuscles, published in the *Deutsche Zeitschrift fur Chirurgie*, and of which a *precis* has appeared in the *British Medical Journal*.

Professor Huter first refers to the changes which the red corpuscles of frog's blood, removed from the body, undergo, on the application of various physical and chemical agents, such as glycerine, ammonia, carbolic acid, chloroform, cold, and heat. These changes, which vary in degree, but are essentially the same in character, consist in notchings and angular irregularities of the corpuscles, often reaching as far as the centre; folding together of the membrane of the corpuscles like a paper envelope; rounded forms are also observed; and sometimes there is nothing left but a nuclear structure, consisting, like the white corpuscles, of fine granules, and distinguishable from the white corpuscles only by its small size and frequently by its oval form. When the above-mentioned agents are applied to the frog's mesentery, this becomes reddened, and microscopic examination shows that the redness depends on dilatation of the vessels combined with a complete stasis of the corpuscles. The idea that this appearance can be explained by the action of the re-agents on the walls of the vessels is contradicted by the rapid occurrence and equally rapid disappearance of the stasis; the phenomenon is rather to be explained by the purely mechanical action of the changes induced in the red corpuscles by means of the various agents, even when applied to the epidermis. Under the microscope the corpuscles can be observed to become indented; first one, and then another, remains hanging on to the walls of the vessel, until at last all the capillaries in the irritated region, and even the adjacent small arteries and veins, are filled with notched red corpuscles (globular stasis). Both during the existence of the stasis, and especially during its rapid breaking up, single altered corpuscles, or large masses of them, may pass into the circulation, and, adhering to the walls of the vessels in other organs, again produce a stasis (globular embolism). This embolism does not lead to the form of distinct embolic plugs, but to diffuse multiplication of stases of corpuscles through the whole circulatory system; for the masses of corpuscles which pass into the circulation readily break up on meeting with any obstacle. The occurrence of embolism from blood-corpuscles in parts where certainly no change in the vessels has occurred, gives a certain degree of support to the assertion that the cause of globular stasis is to be sought in the change of condition of the red corpuscles.

In a pathological point of view, Huter attributes great importance to the stasis of the corpuscles. He believes, in the first place, that irritants act essentially by producing stasis in the region to which they are applied; and that thus the inflammation induced by such means cannot be used in the explanation of the complicated phenomena of ordinary inflammations.

He maintains, further, that these researches are of great importance as regards pharmacology and toxicology. It is already known that many poisons, even those which do not act directly as blood-poisons, produce changes in the blood-corpuscles; and hence it may be supposed that they first produce changes in the form of the corpuscles, then globular stasis, and thus mechanically and indirectly lead to further changes. In experiments on frogs, it was very remarkable that all the agents which induced globular stasis also acted as anæsthetics; this is attributed by Huter to the formation of embola of red corpuscles in the brain. In this way he explains the action of anæsthetics, such as chloroform, ether, and alcohol, the anæsthetic power of which is in proportion to the amount of change of form which they are capable of producing in the red corpuscles. The corpuscles of chloroformed rabbits showed very irregular forms; a notched contour, frequently with one or two large club-shaped processes. After anæsthesia with ether, the corpuscles were mulberry-shaped. After alcohol, there were only slight indentations, and these could be observed in the stage following intoxication both in rabbits and in man. It follows from these observations that the administration of chloroform by inhalation is bad, as in this way the blood-corpuscles are altered while in the lungs, and globular stasis may be produced in these organs. Frogs can be brought into a state of anæsthesia by the action of chloroform - vapor on the skin of the thigh, while at the same time the part where the application is made assumes a redness which cannot be removed by pressure; and the blood-corpuscles removed from this part show the same changes (club-shaped processes, &c.) as in rabbits. This condition is present in the so-called chloroform-erythema which is often observed, especially in tender-skinned individuals. The beneficial action of lowering the head and raising the legs in cases of apparent death from chloroform, may be explained by the more easy breaking up of the globular stasis in this position, as the following experiment shows: The tongue and web of the foot of a chloroformed frog are spread out on a glass plate. If the animal be held for some time with the head upwards, extensive globular stasis will be observed in the vessels of the tongue, while the process is only beginning in the web; when the position is reversed, stasis sets in at once in the web of the foot and disappears from the tongue. Does the weight of the blood in the most dependent parts aid the slightly adherent corpuscles to pass through the vessels? In any case, it seems clear that chloroform is a very dangerous agent, in consequence of its strong action on the blood-corpuscles, and that therefore ether, the action of which is less powerful, is to be preferred as an anæsthetic.

If Huter's explanation of the cause of the anæsthetic action of the substances mentioned be correct, nearly everything which produces globular stasis must also be capable of producing anæsthesia by the formation of globular embolism in the brain; and thus it would seem that even warm baths, perhaps with the addition of easily diffusible agents, might be also used to produce anæsthesia. A frog was immersed for twenty minutes in a five per cent. solution of common salt at the temperature of the room, and was anæsthetized, with the production of globular stasis over the whole skin.

The application of carbolic acid is proper only in the first stages of the treatment of wounds. After the formation of granulations has taken place, it produces permanent stasis of the corpuscles in their vessels, and thus hinders the growth of the granulations as well as the formation of epidermis.

